# Inhibition of Glycogen Synthase Kinase 3β Activity in the Basolateral Amygdala Disrupts Reconsolidation and Attenuates Heroin Relapse

**DOI:** 10.3389/fnmol.2022.932939

**Published:** 2022-06-27

**Authors:** Yuanyang Xie, Yingfan Zhang, Ting Hu, Zijin Zhao, Qing Liu, Haoyu Li

**Affiliations:** ^1^Department of Neurosurgery, Xiangya Hospital, Central South University, Changsha, China; ^2^The Institute of Skull Base Surgery and Neurooncology at Hunan Province, Changsha, China; ^3^Teaching and Research Section of Clinical Nursing, Xiangya Hospital, Central South University, Changsha, China

**Keywords:** addiction, heroin, amygdala, reconsolidation, GSK-3β, self-administration

## Abstract

Exposure to a heroin-associated conditioned stimulus can reactivate drug reward memory, trigger drug cravings, and induce relapse in heroin addicts. The amygdala, a brain region related to emotions and motivation, is involved in processing rewarding stimulus. Recent evidence demonstrated that disrupting the reconsolidation of the heroin drug memories attenuated heroin seeking which was associated with the basolateral amygdala (BLA). Meanwhile, neural functions associated with learning and memory, like synaptic plasticity, are regulated by glycogen synthase kinase 3 beta (GSK-3β). In addition, GSK-3β regulated memory processes, like retrieval and reconsolidation of cocaine-induced memory. Here, we used a heroin intravenous self-administration (SA) paradigm to illustrate the potential role of GSK-3β in the reconsolidation of drug memory. Therefore, we used SB216763 as a selective inhibitor of GSK-3β. We found that injecting the selective inhibitor SB216763 into the BLA, but not the central amygdala (CeA), immediately after heroin-induced memory retrieval disrupted reconsolidation of heroin drug memory and significantly attenuated heroin-seeking behavior in subsequent drug-primed reinstatement, suggesting that GSK-3β is critical for reconsolidation of heroin drug memories and inhibiting the activity of GSK-3β in BLA disrupted heroin drug memory and reduced relapse. However, no retrieval or 6 h after retrieval, administration of SB216763 into the BLA did not alter heroin-seeking behavior in subsequent heroin-primed reinstatement, suggesting that GSK-3β activity is retrieval-dependent and time-specific. More importantly, a long-term effect of SB216763 treatment was observed in a detectable decrease in heroin-seeking behavior, which lasted at least 28 days. All in all, this present study demonstrates that the activity of GSK-3β in BLA is required for reconsolidation of heroin drug memory, and inhibiting GSK-3β activity of BLA disrupts reconsolidation and attenuates heroin relapse.

## Introduction

Opioid use disorder is a chronic recurrent brain disease caused by abnormal learning and memory patterns. And the symptom is the loss of substance use control. Drug-associated cues are important factors to promote the relapse to drug use. In addiction animal models, cues related to drug abuse can promote relapse (Davis and Smith, 1976; Dymshitz and Lieblich, 1987; Di Ciano and Everitt, 2004; Anker and Carroll, 2010). Once drug use occurs, it is possible to form the association between cues and reward-related memory that is not easily disrupted.

Same as other types of memories (Nader et al., 2000; Milekic and Alberini, 2002; Morris et al., 2006; Piva et al., 2019), drug reward memory experiences the process of acquisition, consolidation, retrieval, and reconsolidation. Once a consolidated drug memory is reactivated, it becomes unstable, allowing for modification or destruction by different treatments (Miller and Marshall, 2005; Lee et al., 2006; Wang et al., 2008; Lee, 2009; Li et al., 2010). The role of reconsolidation is critical in stabilizing the reactivated memory, involving de novo protein synthesis (Nader et al., 2000). Reconsolidation provides a time window, during which is expected to modify or even eliminate drug memory. Thus, disrupting reconsolidation of addiction memory is considered to be an effective measure of preventing relapse and drug seeking.

A large amount of evidence shows that pharmacological interventions disrupting reconsolidation of drug-reward memory are effective in relapse prevention. Using a conditioned place preference (CPP) or self-administration (SA) animal model, propranolol or rapamycin has been shown to effectively interfere with reconsolidation of drug memory and attenuate drug-seeking behavior (Lin et al., 2014; Xue et al., 2017; Chen et al., 2021; Zhang et al., 2021).

Glycogen synthase kinase-3 (GSK-3), a serine/threonine-protein kinase expressed widely in the mammalian brain, has essential roles in physiological activities like development, cell cycle, or apoptosis (Jope and Johnson, 2004; Medina et al., 2011). GSK-3β is a isoform of GSK-3, extensively involved in memory processing. The regulation of GSK-3β can affect neural functions like synaptic plasticity, which is the foundation of learning and memory (Banach et al., 2022; Li et al., 2022; Marosi et al., 2022). Also, GSK-3β regulates the structural and functional synaptic plasticity. GSK-3β deficient mice marked that memory reconsolidation and their ability to form long-term memories were impaired, suggesting that GSK-3β is important for normal brain function (O’Brien et al., 2004; Kimura et al., 2008; Kaidanovich-Beilin et al., 2009; Maurin et al., 2013). It has been detected that GSK-3β Ser21/9 phosphorylation levels changed during long-term potentiation (LTP) or long-term depression (LTD), which were essential for memory (Hooper et al., 2007; Peineau et al., 2007). For example, GSK-3β affects long-term memory formation as it promotes LTD by inhibitory phosphorylation of Serine-9 in inhibitory avoidance and novel object recognition test (Dewachter et al., 2009). The removal of GSK-3β in excitatory neurons of dentate gyrus reduced the synaptic transmission of hippocampus and decreased the expression of synaptic proteins like N-methyl-D-aspartate receptor (NMDAR) and anti-alpha-amino-3-hydroxy-5-methyl-4-isoxazolepropinoic receptor (AMPAR), impairing the formation of spatial and fear memories (Liu et al., 2017). Cocaine administration for 14 days could obviously reduce the phosphorylation of GSK-3β in the amygdala (Perrine et al., 2008). Hippocampal GSK-3β was activated during memory retrieval progress in the passive avoidance task (Hong et al., 2012). Memory retrieval activates hippocampal GSK-3β, and memory reconsolidation is impaired by a GSK-3 inhibitor systemically administration before memory retrieval (Kimura et al., 2008; Hong et al., 2012). Knockdown GSK-3β of ventral hippocampal displays a developmental reduction in cocaine-CPP, while not in morphine-CPP (Barr et al., 2020). Wu et al. (2011) directionally inhibited GSK-3β in the BLA immediately after the retrieval of drug cue memories to reduce the subsequent cocaine-seeking behavior (Wu et al., 2011). It has been shown that GSK-3β signaling pathway participated in the reconsolidation of cocaine-induced CPP.

However, whether the inhibition of GSK-3β in BLA could disrupt reconsolidation and prevent drug seeking and relapse in heroin SA animal model remains unknown. In our present study, we inhibited the activity of GSK-3β in the BLA to identify the effects of GSK-3β on reconsolidation of heroin cue memory in SA animal model. Amygdala plays a critical role in both cue-associated learning and the expression of cue-induced relapse of drug-seeking behavior (Luo et al., 2013), and also mediates the reconsolidation of aversive or appetitive memories (McGaugh, 2004; Díaz-Mataix et al., 2013; Nader, 2015; Björkstrand et al., 2016; Haubrich et al., 2020; Higginbotham et al., 2021; Yan et al., 2021). So we chose it as a targeted brain region to deliver SB216763. The role of GSK-3β activity in amygdala on reconsolidation of heroin cue memory was assessed by the heroin self-administration paradigm. More importantly, the long-term inhibitory effects of GSK-3β activity inhibition on reconsolidation of heroin cue memory were also tested.

## Materials and Methods

### Subjects

We housed the Sprague Dawley rats (male, 7-8 weeks of age on arrival) five-per-cage in a 26°C and 60% humidity room and the cycle was 12-h- light/dark (8 a.m.–8 p.m.). Food and water were provided ad libitum. For the animal better adapted to operator, we performed a 5-day grasp-stroking adaptation procedure 3 min per day on experimental animals before the surgeries. All animal procedures and operations were carried out with the approval of the Xiangya Hospital Ethics Committee, Xiangya Hospital (Changsha, China). At the dark phase (8 a.m.–8 p.m.), the experiments were performed.

### Intravenous Surgery

Surgery was performed when the rats’ weight reaches 300–320 g. We used the sodium pentobarbital (60 mg/kg, i.p.) to anesthetize rats. The right jugular was exposed by surgery, then inserted an aseptic catheter into it (Lu et al., 2005; Ambroggi et al., 2008). The catheter passed under the skin of the neck and through the skin of the head and was fixed to the rat’s skull with dental acrylic. Heparinized saline (30 USP heparin/saline; Hospira) was infused into the intravenous catheters per 2 days to prevent clogging. After surgery, rats undergo a 7-day recovery period and their weight should remain constant.

### Cannulae Implantation

Guide cannulae was implanted 1 mm above the BLA or CeA bilaterally after the rats (300–320 g) were anesthetized by using the sodium pentobarbital (60 mg/kg, i.p.). And the coordinate of BLA (Wu et al., 2011) were the following: anterior/posterior: –2.9 mm and medial/lateral: ± 5.0 mm from bregma, dorsal/ventral: –8.5 mm from the surface of the skull. The coordinates for the CeA were the following: anterior/posterior: –2.9 mm and medial/lateral: ± 4.2 mm from bregma, dorsal/ventral: –7.8 mm from the surface of the skull. And the guide cannulae was anchored by the stainless steel screws and dental cement. After surgery, rats undergo a 5–7 days’ recovery period.

### Intracranial Injections

**FIGURE 1:**
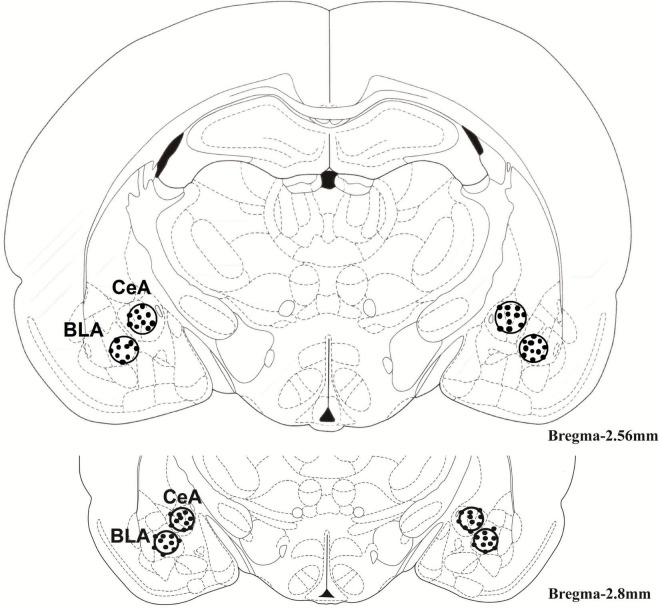
The regions of the basolateral amygdala [basolateral amygdala (BLA): –2.8 mm from bregma] and central amygdala [central amygdala (CeA): –2.8 mm from bregma]. The cannula was placed into them as shown in the rostral faces of each coronal section.

The injection of SB216763 was based on a previous report by some minor changes (Xu et al., 2009). The BLA or CeA (0.5 μL/side) was received drug delivery by a microinjection pump with a rat of 0.5 μL/min. The injection time was no more than one minute. To ensure that the drug is fully administered and fully diffused, wait for more than 1 min to withdraw the needle after administration. Nissl staining was used to verify the cannula placements. A schematic diagram of the injection site in the BLA or CeA is shown in Figure 1.

### Behavioral Procedures

#### Intravenous Heroin Self-Administration (SA) Training

Following the study of Ye et al. (2017), we established an intravenous SA training session experimental conditions were slightly modified. The operant chambers (AniLab Scientific Instruments Co., Ltd., China) used in this experiment were equipped with two nosepoke sensors, which were 5 cm high from the installation floor. The nosepoke sensors could record the number of animals’ nosepokes. The number of nosepoke recorded by the sensor in the left side operandum was defined as “active.” Nosepokes in “active” would lead to intravenous heroin administration with a 5 s tone-light cue synchronization. Corresponding to “active,” the sensor operandum recorded the number of “inactive” nosepoke in the right side. “Inactive” nosepokes had no programmed consequences.

We trained rats to adapt the intravenous heroin self-administration (0.05 mg/kg/infusion) for 10 days. In the training sessions, rats have been trained for three 1-h daily training sessions and every training session was separated by 5 min. The training of rats using a fixed ratio 1 (FR1) reinforcement program up to 40 s. In 10-day training program, rats were received three 1-h heroin infusions intravenously (0.05 mg/kg/infusion) per day, each infusion separated 5 min. All heroin infusions were completed by an injection pump loading a 10 ml syringe. Each training companied a house light illuminated until the end. When rats were at the left nosepoke (active), the maneuver results in an intravenous heroin infusion with a 5 s tone-light cue synchronization. However, the inactive nosepokes had no consequences. To prevent animals’ from death due to overdose, the drug infusions degree was restricted to 20 times/h (Xue et al., 2012; Luo et al., 2015). Some rats (*n* = 11) were excluded from the experiments: five rats died in intravenous surgery and six rats can’t form a stable heroin self-administration.

#### Nosepoke Extinction

After the drug self-administration session, a 3-h daily nosepoke extinction training was followed. In extinction training (Experiments 1–4), the nosepoke behavior of rats to the sensor resulted in no programmed consequences like heroin intravenous delivery, tone-light cues. When at the last three self-administration sessions, the number of active nosepoke responding decreased at least 80% compared with the self-administration, the nosepoke extinction session was ended.

#### Heroin Memory Retrieval Trial

In Experiments 1, 2, and 4, the heroin-associated memories were reacted by performing a 15 min retrieval trial. The conditions of the retrieval session were the same as the SA training session, except that when rats got the “active” sensor, no reward heroin was infusion.

#### Cue Extinction

In Experiments 1, 3, and 4, a cue extinction session, which 3-h per day, was performed on rats. The conditions of the cue extinction session were the same as that SA training session. But after the cue (tone/light) rendering, there was no heroin intravenous delivery.

#### Cue-Induced Reinstatement of Drug-Seeking (Experiments 1–4)

After the SB216763 or vehicle administration (intracranial injections into BLA or CeA), rats got rest for 24 h and then returned to the SA training context and the reinstatement test was performed. In this session, the number of active and inactive nosepokes was recorded for 1 hr. The test condition was the same as the heroin memory retrieval trial.

#### Heroin-Induced Reinstatement Test (Experiments 1, 3, and 4)

A low dose of heroin (0.25 mg/kg, s.c.) was delivered to rats 5 min earlier before the session started, then put rats in the SA training context. Through the two sensors, the number of two type nosepokes (“active” and “inactive”) was recorded during the test. The reinstatement test lasted for 1 h. The test condition was the same as the heroin memory retrieval trial.

#### Spontaneous Recovery Test (Experiment 2)

In this test, after 28 days of the withdrawal phase, two types of nosepokes (“active” and “inactive”) were recorded for 1 h. The test condition was the same as the heroin memory retrieval trial.

### Experimental Design

#### Experiment 1: The Effect of Immediate Post-CS Retrieval SB216763 Treatment on Subsequent Heroin-Seeking Behavior

**FIGURE 2:**
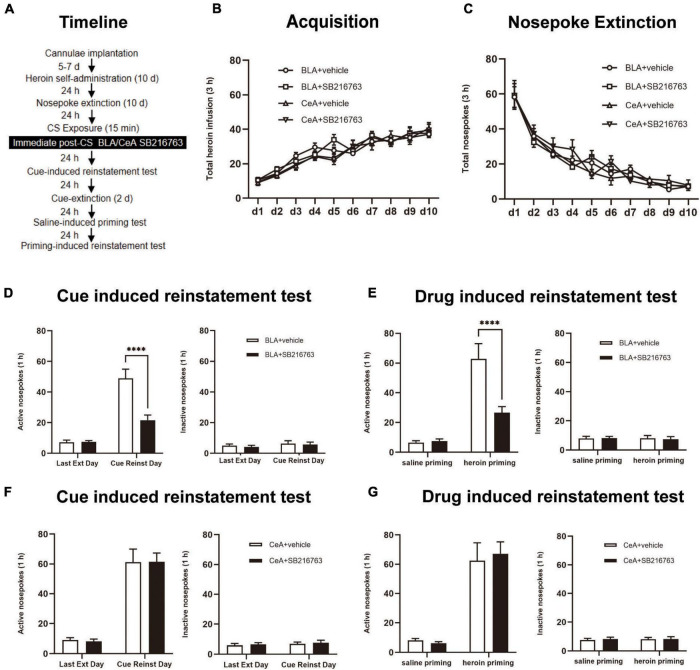
Immediate post-CS SB216763 treatment in BLA rather than CeA can reduce subsequent cue-induced and heroin-primed reinstatement of heroin seeking. **(A)** Timeline of heroin self-administration, nosepoke extinction, cue-induced reinstatement test and drug-induced reinstatement test. **(B)** Total number of heroin infusions during acquisition of heroin self-administration. **(C)** Total number of active nosepoke responses during extinction training sessions. **(D,F)** Active (left) and inactive (right) nosepoke responses during the last extinction training session and the cue-induced reinstatement test. **(D)** Nosepoke responses of rats with BLA drug injection. **(F)** Nosepoke responses of rats with CeA drug injection. **(E,G)** Active (left) and inactive (right) nosepoke responses across the saline- or heroin- primed reinstatement test. **(E)** Nosepoke responses of rats with BLA drug injection. **(G)** Nosepoke responses of rats with CeA drug injection. *n* = 10–11 mice per group. Data are means ± SEM, ^****^p < 0.0001, compared with the vehicle group. CS, conditioned stimulus; Ext, extinction; Reinst, reinstatement.

Through 10 days of heroin SA training, nosepoke extinction training was followed in the same apparatus for 10 consecutive days. After nosepoke extinction training, rats were allowed a rest for 24 h, then rats were received a 15-min conditioned stimulus (CS) retrieval session. After CS exposure, the rats were divided into four groups: (1) Intracranial injection of vehicle into BLA (0.5 μL/side) immediate after the retrieval trial (BLA+vehicle); (2) Intracranial injection of SB216763 into BLA (0.5 μL/side) immediate after a 15 min retrieval test (BLA+SB216763); (3) Intracranial injection of vehicle into CeA (0.5 μL/side) immediate after a 15 min retrieval test (CeA+vehicle); (4) Intracranial injection of SB216763 into CeA (0.5 μL/side) immediate after a 15 min retrieval test (CeA+SB216763). On Day 23, the rats were performed a cue-induced reinstatement test to explore the effect of SB216763 on heroin drug memory. Subsequently, the two-day cue extinction session was carried out. On Day 26, heroin priming-induced reinstatement was tested in rats (Figure 2A).

#### Experiment 2: The Lasting and Long-Term Effect of Immediate Post-CS Retrieval SB216763 Treatment on Cue-Induced Reinstatement Test and Spontaneous Recovery 28 Days Later

**FIGURE 3:**
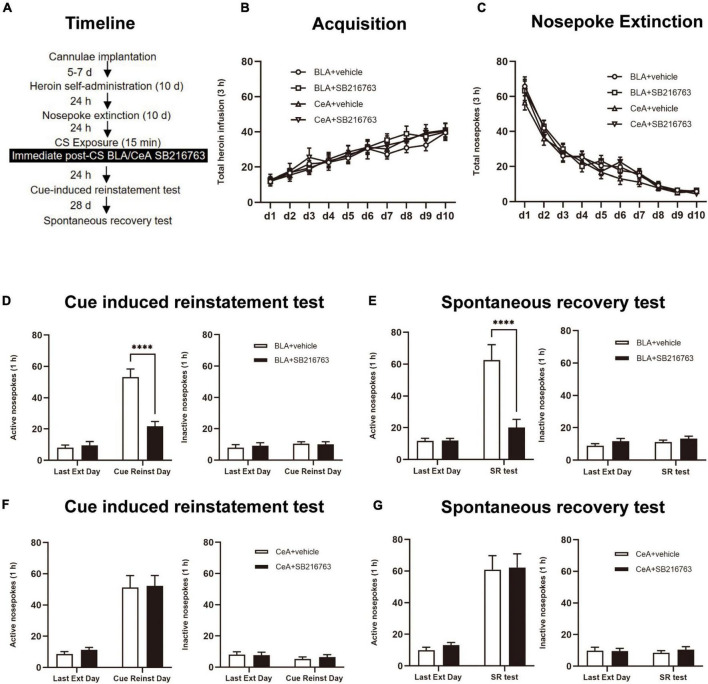
Immediate post-CS SB216763 treatment in BLA rather than CeA can reduce subsequent cue-induced heroin seeking and the spontaneous recovery of heroin seeking. **(A)** Timeline of heroin self-administration, nosepoke extinction, cue-induced reinstatement test and spontaneous recovery test. **(B)** Total number of heroin infusions during acquisition of heroin self-administration. **(C)** Total number of active nosepoke responses during extinction training sessions. **(D,F)** Active (left) and inactive (right) nosepoke responses during the last extinction training session and the cue-induced reinstatement test. **(D)** Nosepoke responses of rats with BLA drug injection. **(F)** Nosepoke responses of rats with CeA drug injection. **(E,G)** Active (left) and inactive (right) nosepoke responses across the last extinction training session and spontaneous recovery test. **(E)** Nosepoke responses of rats with BLA drug injection. **(G)** Nosepoke responses of rats with CeA drug injection. n = 10 mice per group. Data are means ± SEM, ^****^p < 0.0001, compared with the vehicle group. CS, conditioned stimulus; Ext, extinction; Reinst, reinstatement; SR, spontaneous recovery.

After CS exposure, the rats were divided into four groups: (1) Intracranial administration of vehicle into BLA (0.5 μL/side) immediate after a 15 min retrieval trial (BLA+vehicle); (2) Intracranial injection of SB216763 into BLA (0.5 μL/side) immediate after a 15 min retrieval trial (BLA+SB216763); (3) Intracranial injection of the vehicle into CeA (0.5 μL/side) immediate after a 15 min retrieval trial (CeA+vehicle); (4) Intracranial injection of SB216763 into CeA(0.5 μL/side) immediate after a 15 min retrieval (CeA+SB216763). Rats were received a cue-induced reinstatement test after a 24 h rest. After 28 days of abstinence, to access the long-term effect of SB216763 on heroin-seeking behavior, a spontaneous recovery test was carried out (Figure 3A).

#### Experiments 3 A and B: The Effect of SB216763 Treatment on Subsequent Heroin-Seeking Behavior Without CS Exposure

**FIGURE 4:**
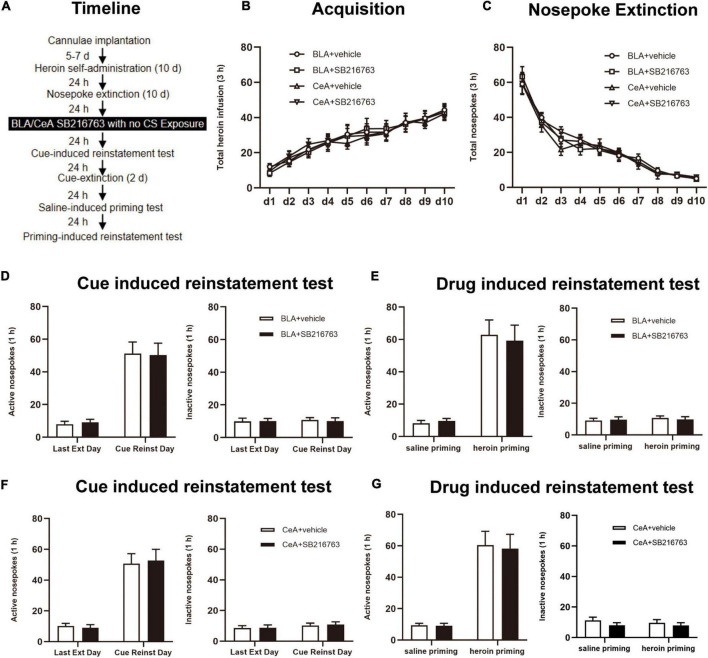
SB216763 treatment without CS retrieval does not affect subsequent cue-induced and heroin-primed reinstatement of heroin seeking. **(A)** Timeline of heroin self-administration, nosepoke extinction, cue-induced reinstatement test and drug-induced reinstatement test. **(B)** Total number of heroin infusions during acquisition of heroin self-administration. **(C)** Total number of active nosepoke responses during extinction training sessions. **(D,F)** Active (left) and inactive (right) nosepoke responses during the last extinction training session and the cue-induced reinstatement test. **(D)** Nosepoke responses of rats with BLA drug injection. **(F)** Nosepoke responses of rats with CeA drug injection. **(E,G)** Active (left) and inactive (right) nosepoke responses across the saline- or heroin- primed reinstatement test. **(E)** Nosepoke responses of rats with BLA drug injection. **(G)** Nosepoke responses of rats with CeA drug injection. *n* = 10 mice per group. Data are means ± SEM. CS, conditioned stimulus; Ext, extinction; Reinst, reinstatement.

The experimental procedure was the same as Experiment 1, except that no CS exposure session to reactivate heroin cue memories (Figure 4A).

#### Experiment 4: The Effect of Delayed Post-CS Retrieval SB216763 Treatment

**FIGURE 5:**
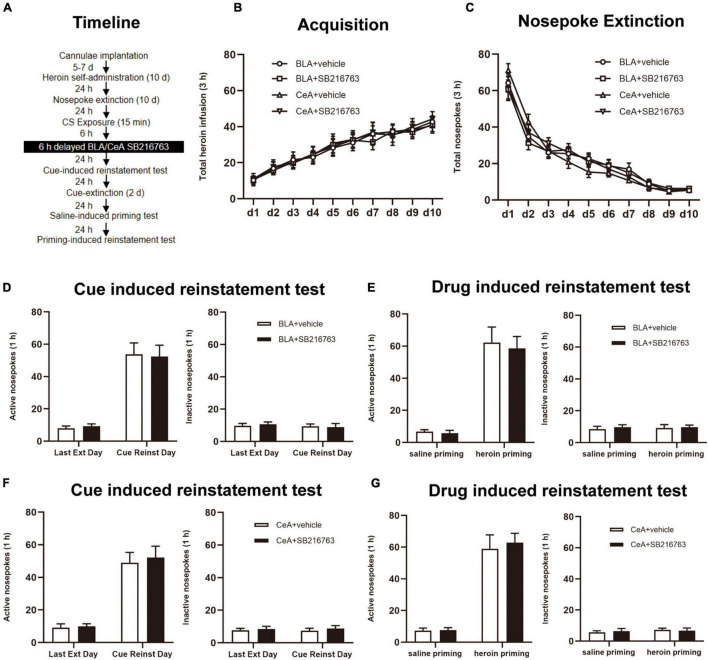
SB216763 treatment 6 h after retrieval does not affect the heroin seeking in the following cue-induced and heroin-primed reinstatement. **(A)** Timeline of heroin self-administration, nosepoke extinction, cue-induced reinstatement test and drug-induced reinstatement test. **(B)** Total number of heroin infusions during acquisition of heroin self-administration. **(C)** Total number of active nosepoke responses during extinction training sessions. **(D,F)** Active (left) and inactive (right) nosepoke responses during the last extinction training session and the cue-induced reinstatement test. **(D)** Nosepoke responses of rats with BLA drug injection. **(F)** Nosepoke responses of rats with CeA drug injection. **(E,G)** Active (left) and inactive (right) nosepoke responses across the saline- or heroin- primed reinstatement test. **(E)** Nosepoke responses of rats with BLA drug injection. **(G)** Nosepoke responses of rats with CeA drug injection. *n* = 9–10 mice per group. Data are means ± SEM. CS, conditioned stimulus; Ext, extinction; Reinst, reinstatement.

The rats were received treatment of SB216763 delayed for 6 h after the retrieval session, and other parts of the experiment were the same as Experiment 1 (Figure 5A).

### Statistical Analysis

We used the repeated-measures ANOVAs to analyze the data with the between-subjects factor of treatment condition and within-subjects factor of test condition, followed by Tukey’s *post-hoc test* in each experiment (see Results). All values of experiments were presented as mean ± SEM and data analysis was done on GraphPad, v.9.0. *p* values < 0.05 were considered statistically significant.

## Results

### Experiment 1: Immediate Post-CS SB216763 Treatment in BLA Rather Than CeA Can Reduce Subsequent Cue-Induced and Heroin-Primed Reinstatement of Heroin Seeking

We tested the influences of post-retrieval BLA and CeA SB216763 injection on reinstatement of heroin seeking which was cue-induced or heroin-induced by using four groups of rats (Figure 2A). In the acquisition session, there were no significant differences between groups of BLA injected with vehicle (*N* = 10) or SB216763 (N = 11) and groups of CeA treated with vehicle (N = 10) or SB216763 (N = 11) which were shown by the heroin infusion numbers [main effect of acquisition time: F_(9,342)_ = 81.35, *p* < 0.0001; administration condition: F_(3, 38)_ = 0.2803, *p* = 0.8393; acquisition time × administration condition interaction: F_(27, 342)_ = 1.443, *p* = 0.0744; Figure 2B]. As the numbers of nosepokes showed, groups did not significantly differ from each other in the extinction session [main effect of extinction time: F_(9, 342)_ = 95.68, *p* < 0.0001; administration condition: F_(3, 38)_ = 0.092, *p* = 0.9640; extinction time × administration condition interaction: F_(27, 342)_ = 0.8897, *p* = 0.6274; Figure 2C].

In active nosepokes, BLA+vehicle and BLA+SB216763 groups were significantly different in cue-induced reinstatement test [main effect of trial condition: F_(1, 37)_ = 65.71, *p* < 0.0001; administration condition: F_(1, 37)_ = 15.47, *p* = 0.0004; trial condition × administration condition interaction: F_(1, 37)_ = 16.05, *p* = 0.0003]. In cue-induced reinstatement test, the *post-hoc* test revealed a reduction significantly in active side nosepokes of BLA+SB216763 group compared to that BLA+vehicle (*p* < 0.001) (Figure 2D left), while there was no obvious difference in the inactive side [main effect of trial condition: F_(1, 19)_ = 1.187, *p* = 0.2895; administration condition: F_(1, 19)_ = 0.2169, *p* = 0.6467; trial condition × administration condition interaction: F_(1, 19)_ = 0.0156, *p* = 0.9020; Figure 2D right]. While in the cue-induced reinstatement test of the CeA+vehicle and CeA+SB216763 groups, no significant differences were found in active side [main effect of trial condition: F_(1, 38)_ = 101.4, *p* < 0.0001; administration condition: F_(1, 38)_ = 0.0052, *p* = 0.9432; trial condition × administration condition interaction: F_(1, 38)_ = 0.0140, *p* = 0.9063; Figure 2F left] and the inactive side [main effect of trial condition: F_(1, 19)_ = 0.7022, *p* = 0.4125; administration condition: F_(1, 19)_ = 0.1727, *p* = 0.6824; trial condition × administration condition interaction: F_(1, 19)_ = 0.0065, *p* = 0.9368; Figure 2F right]. In addition, in drug-induced reinstatement test, active nosepokes significantly differed in BLA+vehicle and BLA+SB216763 groups [main effect of trial condition: F_(1, 19)_ = 51.16, *p* < 0.0001; administration condition: F_(1, 19)_ = 9.702, *p* = 0.0057; trial condition × administration condition interaction: F_(1, 19)_ = 12.55, *p* = 0.0022]; the *post-hoc* test showed a significant reduction of BLA+SB216763 group in drug-seeking compared with BLA+vehicle group in heroin priming-induced reinstatement test (*p* < 0.0001) (Figure 2E left), but did not differ in the inactive side [main effect of trial condition: F_(1, 19)_ = 0.0450, *p* = 0.8342; administration condition: F_(1, 19)_ = 0.0123, *p* = 0.9130; trial condition × administration condition interaction: F_(1, 19)_ = 0.0736, *p* = 0.7891; Figure 2E right]. Moreover, no significant differences were found in active side of CeA+vehicle and CeA+SB216763 groups in drug-induced reinstatement test [main effect of trial condition: F_(1, 19)_ = 67.29, *p* < 0.0001; administration condition: F_(1, 19)_ = 0.0369, *p* = 0.8498; trial condition × administration condition interaction: F_(1, 19)_ = 0.2259, *p* = 0.6400; Figure 2G left column] or the inactive side [main effect of trial condition: F_(1, 19)_ = 0.0218, *p* = 0.8841; administration condition: F_(1, 19)_ = 0.1738, *p* = 0.6814; trial condition × administration condition interaction: F_(1, 19)_ = 0.0218, *p* = 0.8841; Figure 2G right].

The results suggest that injecting SB216763 in BLA but not the CeA reduced cue-induced and heroin-primed heroin seeking reinstatement immediately after heroin cue retrieval.

### Experiment 2: Immediate Post-CS SB216763 Treatment in BLA Rather Than CeA Can Reduce Subsequent Cue-Induced Heroin Seeking and the Spontaneous Recovery of Heroin Seeking

We tested the influences of immediate post-retrieval SB216763 injection in BLA and CeA on heroin seeking reinstatement which was cue-induced or heroin-induced besides the influences of long-term by using four rats groups (Figure 3A). In the heroin self-administration acquisition session, groups of BLA administrated vehicle (*N* = 10) or SB216763 (*N* = 10) and of CeA administrated vehicle (*N* = 10) or SB216763 (*N* = 10) had no significant differences that were shown by the similar heroin infusion numbers [main effect of acquisition time: F_(9, 324)_ = 48.79, *p* < 0.0001; administration condition: F_(3, 36)_ = 0.1662, *p* = 0.9185; acquisition time × administration condition interaction: F_(27, 324)_ = 0.5552, *p* = 0.9663; Figure 3B]. Analogously, no significant differences of group were found in 10-day extinction training [main effect of extinction time: F_(9, 324)_ = 207.2, *p* < 0.0001; administration condition: F_(3, 36)_ = 0.5019, *p* = 0.6833; extinction time × administration condition interaction: F_(27, 324)_ = 0.9528, *p* = 0.5354; Figure 3C].

Consistent with the results of Experiment 1, in the cue-induced reinstatement test, we found that nosepokes of groups differed from each other significantly in active side of BLA+vehicle and BLA+SB216763 groups [main effect of trial condition: F_(1, 18)_ = 67.92, *p* < 0.0001; administration condition: F_(1, 18)_ = 21.07, *p* = 0.0002; trial condition × administration condition interaction: F_(1, 18)_ = 22.67, *p* = 0.0002]. In the cue-induced reinstatement test, the *post-hoc* test shown a significant reduction in active side nosepokes of BLA+SB216763 group compared to that BLA+vehicle (*p* < 0.001) (Figure 3D left), but not in the inactive side [main effect of trial condition: F_(1, 18)_ = 1.212, *p* = 0.2854; administration condition: F_(1, 18)_ = 0.0327, *p* = 0.8585; trial condition × administration condition interaction: F_(1, 18)_ = 0.2860, *p* = 0.5993; Figure 3D right]. And in the cue-induced reinstatement test, there were no significant differences between CeA+vehicle and CeA+SB216763 groups of the active side [main effect of trial condition: F_(1, 18)_ = 53.73, *p* < 0.0001; administration condition: F_(1, 18)_ = 0.1503, *p* = 0.7028; trial condition × administration condition interaction: F_(1, 18)_ = 0.0223, *p* = 0.8830; Figure 3F left column; Figure 3F left] and the inactive side [main effect of trial condition: F_(1, 18)_ = 1.683, *p* = 0.2110; administration condition: F_(1, 18)_ = 0.0485, *p* = 0.8281; trial condition × administration condition interaction: F_(1, 18)_ = 0.3407, *p* = 0.5666; Figure 3F right]. Furthermore, during the spontaneous recovery test, active nosepokes significantly differed in BLA+vehicle and BLA+SB216763 groups [main effect of trial condition: F_(1, 18)_ = 29.44, *p* < 0.0001; administration condition: F_(1, 18)_ = 13.79, *p* = 0.0016; trial condition × administration condition interaction: F_(1, 18)_ = 15.39, *p* = 0.0010]. During the spontaneous recovery test, a *post-hoc* test showed a significant reduction of BLA+SB216763 group in drug-seeking compared with BLA+vehicle group *(p* < 0.0001) (Figure 3E left) but not in the inactive side [main effect of trial condition: F_(1, 18)_ = 1.688, *p* = 0.2103; administration condition: F_(1, 18)_ = 2.840, *p* = 0.1092; trial condition × administration condition interaction: F_(1, 18)_ = 0.0604, *p* = 0.8086; Figure 3E right]. While, in spontaneous recovery test, no significant differences of CeA+vehicle and CeA+SB216763 groups were found in active side [main effect of trial condition: F_(1, 18)_ = 74.83, *p* < 0.0001; administration condition: F_(1, 18)_ = 0.1074, *p* = 0.7469; trial condition × administration condition interaction: F_(1, 18)_ = 0.027,*p* = 0.8714; Figure 3G left] and the inactive side [main effect of trial condition: F_(1, 18)_ = 0.0110, *p* = 0.9176; administration condition: F_(1, 18)_ = 0.1554, *p* = 0.6981; trial condition × administration condition interaction: F_(1, 18)_ = 0.6468, *p* = 0.4318; Figure 3G right].

Thus, the results suggest that immediate post-CS BLA SB216763 treatment rather than CeA can reduce subsequent cue-induced heroin seeking and last at least 28 days.

### Experiment 3: SB216763 Treatment Without CS Retrieval Does Not Affect Subsequent Cue-Induced and Heroin-Primed Reinstatement of Heroin Seeking

We tested whether SB216763 affecting subsequent heroin seeking depends on CS retrieval by using four rats groups. When heroin acquisition and extinction training were finished, rats were received BLA or CeA SB216763 treatment with no CS exposure (Figure 4A). During the training sessions of heroin self-administration, groups of BLA administrated vehicle (*N* = 10) or SB216763 (*N* = 10) and of CeA administrated vehicle (*N* = 10) or SB216763 (*N* = 10) had no differences in acquisition session [main effect of acquisition time: F_(9, 324)_ = 37.72, *p* < 0.0001; administration condition: F_(3, 36)_ = 0.0764, *p* = 0.9724; acquisition time × administration condition interaction: F_(27, 324)_ = 0.1869, *p* > 0.9999; Figure 4B] and in 10-day extinction training [main effect of extinction time: F_(9, 324)_ = 127.0, *p* < 0.0001; administration condition: F_(3, 36)_ = 0.1659, *p* = 0.9187; extinction time × administration condition interaction: F_(27, 324)_ = 0.4249, *p* = 0.9953; Figure 4C] as suggested by the similar heroin infusions.

Cue-induced reinstatement test was shown that no significant differences were found in nosepokes of active side [main effect of trial condition: F_(1, 18)_ = 67.15, *p* < 0.0001; administration condition: F_(1, 18)_ = 0.0008, *p* = 0.9778; trial condition × administration condition interaction: F_(1, 18)_ = 0.0415, *p* = 0.8409; Figure 4D, left] and inactive side [main effect of trial condition: F_(1, 18)_ = 0.0816, *p* = 0.7784; administration condition: F_(1, 18)_ = 0.0184, *p* = 0.8937; trial condition × administration condition interaction: F_(1, 18)_ = 0.0816, *p* = 0.7784; Figure 4D, right] between the BLA+vehicle and BLA+SB216763 groups. Similarly, no significant differences of nosepokes were found in active side [main effect of trial condition: F_(1, 18)_ = 78.74, *p* < 0.0001; administration condition: F_(1, 18)_ = 0.0068, *p* = 0.9353; trial condition × administration condition interaction: F_(1, 18)_ = 0.1212, *p* = 0.7317; Figure 4F, left] or in inactive side [main effect of trial condition: F_(1, 18)_ = 1.561, *p* = 0.2275; administration condition: F_(1, 18)_ = 0.0312, *p* = 0.8618; trial condition × administration condition interaction: F_(1, 18)_ = 0.0285, *p* = 0.8678; Figure 4F, right] of CeA+vehicle and CeA+SB216763 groups. In drug-induced reinstatement test, no significant differences of nosepokes were found in active side [main effect of trial condition: F_(1, 18)_ = 61.05, *p* < 0.0001; administration condition: F_(1, 18)_ = 0.0232, *p* = 0.8806; trial condition × administration condition interaction: F_(1, 18)_ = 0.1465, *p* = 0.7064; Figure 4E, left] and in inactive side [main effect of trial condition: F_(1, 18)_ = 0.3709, *p* = 0.5501; administration condition: F_(1, 18)_ = 0.0075, *p* = 0.9318; trial condition × administration condition interaction: F_(1, 18)_ = 0.2887, *p* = 0.5976; Figure 4E, right] in BLA+vehicle and BLA+SB216763 groups. Besides, it was not significantly different in nosepokes of active side [main effect of trial condition: F_(1, 18)_ = 58.06, *p* < 0.0001; administration condition: F_(1, 18)_ = 0.0397, *p* = 0.8444; trial condition × administration condition interaction: F_(1, 18)_ = 0.0209, *p* = 0.8866; Figure 4G, left] or inactive side [main effect of trial condition: F_(1, 18)_ = 0.1848, *p* = 0.6724; administration condition: F_(1, 18)_ = 2.064, *p* = 0.1679; trial condition × administration condition interaction: F_(1, 18)_ = 0.1118, *p* = 0.7420; Figure 4G, right] between the CeA+vehicle and CeA+SB216763 groups.

Thus, the results indicate that the CS retrieval of drug-associated memory determines the effect of SB216763 on subsequent heroin seeking and when SB216763 is used alone, there will be no influence on the heroin seeking of the subsequent cue-induced and heroin-primed reinstatement test.

### Experiment 4: SB216763 Treatment 6 h After Retrieval Does Not Affect the Heroin Seeking in the Following Cue-Induced and Heroin-Primed Reinstatement

Finally, we employed whether SB216763 treated out of the reconsolidation time window would suppress the following heroin seeking (Figure 5A). During the training sessions, groups of BLA administrated vehicle (*N* = 10) or SB216763 (*N* = 10) and of CeA administrated vehicle (*N* = 10) or SB216763 (*N* = 10) had no differences in heroin self-administration acquisition [main effect of acquisition time: F_(9, 315)_ = 32.37, *p* < 0.0001; administration condition: F_(3, 35)_ = 0.0423, *p* = 0.9882; acquisition time × administration condition interaction: F_(27, 315)_ = 0.1182, *p* > 0.9999; Figure 5B] and in 10-day extinction sessions [main effect of extinction time: F_(9, 315)_ = 155.5, *p* < 0.0001; administration condition: F_(3, 35)_ = 0.0613, *p* = 0.9798; extinction time × administration condition interaction: F_(27, 315)_ = 1.120, *p* = 0.3139; Figure 5C] which were shown by the similar heroin injections numbers.

In cue-induced reinstatement test, the nosepokes did not significantly differ in the active side [main effect of trial condition: F_(1, 18)_ = 73.20, *p* < 0.0001; administration condition: F_(1, 18)_ = 0.0001, *p* = 0.9922; trial condition × administration condition interaction: F_(1, 18)_ = 0.0675, *p* = 0.7979; Figure 5D, left] or in the inactive side [main effect of trial condition: F_(1, 18)_ = 0.3240, *p* = 0.5763; administration condition: F_(1, 18)_ = 0.0135, *p* = 0.9088; trial condition × administration condition interaction: F_(1, 18)_ = 0.2073, *p* = 0.6543; Figure 5D, right] of BLA+vehicle and BLA+SB216763 groups. Similarly, no significant differences in nosepokes were found in active side [main effect of trial condition: F_(1, 17)_ = 67.32, *p* < 0.0001; administration condition: F_(1, 18)_ = 0.1421, *p* = 0.7106; trial condition × administration condition interaction: F_(1, 17)_ = 0.0610, *p* = 0.8079; Figure 5F, left] or in the inactive side [main effect of trial condition: F_(1, 17)_ = 0.0001, *p* = 0.9944; administration condition: F_(1, 17)_ = 0.3902, *p* = 0.5405; trial condition × administration condition interaction: F_(1, 17)_ = 0.0182, *p* = 0.8943; Figure 5F, right] of CeA+vehicle and CeA+SB216763 groups. As for the drug-induced reinstatement test, the nosepokes did not significantly differ in the active side [main effect of trial condition: F_(1, 18)_ = 77.38, *p* < 0.0001; administration condition: F_(1, 18)_ = 0.1286, *p* = 0.7241; trial condition × administration condition interaction: F_(1, 18)_ = 0.0481, *p* = 0.8289; Figure 5E, left] or in the inactive side [main effect of trial condition: F_(1, 18)_ = 0.0473, *p* = 0.8304; administration condition: F_(1, 18)_ = 0.2022, *p* = 0.6583; trial condition × administration condition interaction: F_(1, 18)_ = 0.0241, *p* = 0.8783; Figure 5E, right] between the BLA+vehicle and BLA+SB216763 groups. Besides, the nosepokes did not significantly differ in the active side [main effect of trial condition: F_(1, 17)_ = 94.30, *p* < 0.0001; administration condition: F_(1, 18)_ = 0.1377, *p* = 0.7149; trial condition × administration condition interaction: F_(1, 17)_ = 0.0955, *p* = 0.7611; Figure 5G, left] or in the inactive side [main effect of trial condition: F_(1, 17)_ = 0.3672, *p* = 0.5525; administration condition: F_(1, 17)_ = 0.0108, *p* = 0.9184; trial condition × treatment condition interaction: F_(1, 17)_ = 0.2189, *p* = 0.6458; Figure 5G, right] in the CeA+vehicle and CeA+SB216763 groups.

The results suggest that the effect of SB216763 on the heroin seeking of the following cue-induced and heroin-primed reinstatement test is time-specific.

## Discussion

In our present study, we examined the effects of SB216763, a GSK-3β inhibitor, on heroin-seeking behavior and relapse *via* an intravenous heroin SA procedure. The main findings are following: (1) Immediate post-CS SB216763 treatment in BLA rather than CeA can reduce subsequent cue-induced and heroin-primed reinstatement of heroin seeking. (2) The effect of SB216763 (intracranial injection into BLA) immediately after retrieval session, which reactivated the heroin cue memory, on cocaine-seeking behavior continued for at least twenty-eight days; (3) SB216763 administration into BLA delayed 6 h or without the retrieval have no effect on the cue-induced and heroin priming-induced reinstatement test, suggesting that the effects of SB216763 on drug-seeking behavior are retrieval-dependent and time-specific. The results demonstrated that the activity of GSK-3β in BLA is required for reconsolidation and intra-BLA injection of SB216763 disrupts memory reconsolidation, suggested that inhibiting GSK-3β activity of BLA disrupts reconsolidation and attenuates heroin relapse.

Existing evidence has shown that consolidated drug-induced memory could be unstable after retrieval by conditioned stimulus (drug-associated cues) or unconditioned stimulus (the drugs). Disrupting the reconsolidation after drug memory retrieval effectively blocks the association between drug-related cues and drugs, thus producing a forgetting effect and reducing drug-seeking behavior. Memory retrieval activates GSK-3β in the BLA, and reconsolidation was impaired by systemical administration of a GSK-3 inhibitor before memory retrieval in rats (Wu et al., 2011). We have demonstrated that SB216763, as a GSK-3β inhibitor, reduced heroin seeking and relapse by disrupting reconsolidation of heroin drug memory. Nevertheless, without CS exposure or with a 6h delay administration of SB216763 has no effect on heroin seeking behavior, which is consistent with the “reconsolidation theory” that allowing for modifications during reconsolidation in a putative time window. Indeed, GSK-3β expressed in multiple brain regions (e.g., NAc, PFC, amygdala) (Winder et al., 2002; Morgane et al., 2005; Robbins et al., 2008), regulates the dopamine-associated behaviors (Beaulieu et al., 2007a). The role of GSK-3β in drug addiction has also been confirmed by several studies. For example, in a cocaine-induced CPP model, the reconsolidation of cocaine-cue memories was impaired by systemic injection of lithium chloride (a GSK-3β inhibitor) after memory retrieval, and SB216763 administration into BLA can result in a long-term effect on cocaine cue memories *via* disrupting reconsolidation (Wu et al., 2011).

Our present study complements the role of GSK-3β in heroin self-administration model. We show that inhibiting GSK-3β activity by injection of SB216763 into the BLA, but not the CeA, immediately after the retrieval reduced the drug-seeking behavior. In addition, some studies have shown that the GSK-3β in the hippocampus regulates learning and memory ability. Targeted downregulation of GSK-3β in the ventral hippocampus disrupting the formation of cocaine CPP and the spatial memory (Barr et al., 2020). Overexpression of GSK-3β in the hippocampus impaired the spatial memory (Liu et al., 2020). And both the amygdala, hippocampus, are the important brain regions in drug addiction (Alizamini et al., 2022). Previous study found that inhibited the activity of GSK-3β in the BLA immediately after the retrieval could disrupte cocaine context memory (Wu et al., 2011). Our findings are consistent with these results and showed that inhibiting GSK-3β in the BLA in the reconsolidation time window decreased drug seeking and relapse in heroin SA rats.

The amygdala, associated with emotion and motivation, plays roles in processing rewarding environmental stimulus (Janak and Tye, 2015). Recent evidence demonstrates that disrupting the reconsolidation of the cocaine drug memories attenuates cocaine seeking which was associated with the BLA (Wu et al., 2011). In our present study, we discussed the role of GSK-3β in a heroin self-administration rat model. Inhibiting activity of GSK-3β in the BLA, but not the CeA, effectively disrupted reconsolidation of heroin cue memory and blocked subsequent drug-seeking behaviors. Many studies have demonstrated that BLA is involved in memory reconsolidation (Wells et al., 2013; Yuan et al., 2020). However, the spcefic mechanism of GSK-3β in drug memory reconsolidation remains to be elucidated.

GSK-3β is widely expressed in the brain and involved in fundamental brain functions like neurogenesis, neurotransmitter signaling (Beaulieu et al., 2004, 2007b; Li et al., 2004), circadian rhythms (Yin et al., 2006), and memory process (Hooper et al., 2007; Kimura et al., 2008; Hong et al., 2012). GSK-3β has been shown to regulate many transcription factors (e.g., β-catenin, NF-κB, activator protein-1), and memory-associated proteins which have been implicated in fundamental brain functions (Frame and Cohen, 2001). Specifically, in Liu et al.’s study, the role of GSK-3β/β-catenin signaling pathway on memory consolidation in rats trained in a Morris water maze task was proved (Liu et al., 2014) and the consolidation of fear memory need the β-catenin in the amygdala (Maguschak and Ressler, 2008). As a study revealed, the Akt/GSK-3/mTORC1 signaling pathway, which has been found in the hippocampus and nucleus accumbens, participates in the reconsolidation progress of cocaine memory (Shi et al., 2014). Psychostimulant activates GSK-3β by inactivating protein kinase B (Akt) and reducing its inhibition of serine-phosphorylation (Miller et al., 2014). These findings indicate that during the reconsolidation process, GSK-3β may be required to be activated, but the role of GSK-3β on reconsolidation of heroin-SA and the possible molecular mechanisms involved in this process have not yet known. Our present study showed that intra-BLA infusion of the GSK-3β inhibitor SB216763 disrupted the reconsolidation of heroin reward memory and the reduction of heroin seeking behaviors lasted for a long time (28 days). And these effects were BLA- specific and time-specific. Studies have reported that the activity of GSK-3β was involved in the LTP and LTD which both depended on the NMDA receptor and also regulated the interreaction between them (Peineau et al., 2007, 2008, 2009). But there is less study that reported the relationship between GSK-3β and NMDA in addiction. Furthermore, reconsolidation is associated with de novo protein synthesis and synaptic plasticity alteration (Nader et al., 2000). Thus, our future study will focus on the relationship between GSK-3β and NMDA in reconsolidation of heroin reward memory and investigate the synaptic plasticity alteration during this process by electrophysiological methods. Compared with Wu et al. (2011) study, our present study is the extension of previous studies, and we used a drug self-administration paradigm, which can simulate craving and relapse in addicts and is more suitable for studying craving and relapse (Spealman and Goldberg, 1978; Schindler et al., 2002).

In summary, the present study demonstrated the effects of GSK-3β on the reconsolidation of drug reward memory *via* a rats heroin SA paradigm. After heroin cue memory retrieval, injection of SB216763 into BLA impairs reconsolidation of the heroin cue memory, effectively reduces the heroin seeking behavior in rats, and these effects lasted at least 28 days. Moreover, the reconsolidation window may be an important determinant to reduce heroin seeking and relapse. Our present study identifies that GSK-3β inhibitors may have the potential therapeutic value in the treatment of heroin addiction.

## Data Availability Statement

The raw data supporting the conclusions of this article will be made available by the authors, without undue reservation.

## Ethics Statement

The animal study was reviewed and approved by the Xiangya Hospital Ethics Committee, Xiangya Hospital (Changsha, China).

## Author Contributions

HL and YX: conceptualization. YZ and YX: data curation. TH and ZZ: writing – original draft preparation. YX and QL: writing – review and editing. HL: supervision and funding acquisition. All authors have read and agreed to the published version of the manuscript.

## Conflict of Interest

The authors declare that the research was conducted in the absence of any commercial or financial relationships that could be construed as a potential conflict of interest.

## Publisher’s Note

All claims expressed in this article are solely those of the authors and do not necessarily represent those of their affiliated organizations, or those of the publisher, the editors and the reviewers. Any product that may be evaluated in this article, or claim that may be made by its manufacturer, is not guaranteed or endorsed by the publisher.
